# Genome Sequence of a Novel Positive-Sense, Single-Stranded RNA Virus Isolated from Western Corn Rootworm, *Diabrotica virgifera virgifera* LeConte

**DOI:** 10.1128/genomeA.00366-17

**Published:** 2017-05-18

**Authors:** Sijun Liu, Yuting Chen, Thomas W. Sappington, Bryony C. Bonning

**Affiliations:** a Department of Entomology, Iowa State University, Ames, Iowa, USA; b Corn Insects and Crop Genetics Research Unit, USDA, ARS, Ames, Iowa, USA

## Abstract

The genome sequence of a novel small RNA virus was assembled from the transcriptome of the western corn rootworm, *Diabrotica virgifera virgifera*. The assembled genome has 13,182 nucleotides with a 3′ polyadenylated tail. Two open reading frames are predicted to encode polyproteins of 2,838 and 1,073 amino acids.

## GENOME ANNOUNCEMENT

Western corn rootworm (WCR), *Diabrotica virgifera virgifera* LeConte (*Coleoptera*: *Chrysomelidae*), causes significant yield losses in *Zea mays* L. in North America and Europe. In efforts to devise novel virus-based management approaches for this pest, we investigated the viral pathogens of WCR. Adult and larval WCR were collected from the United States and Europe, and RNA was isolated from ~100 whole insects per location, or from viruses purified from the WCR samples as described previously (1). RNA sequencing (RNA-Seq) libraries were prepared using the TruSeq RNA sample preparation kit version 2 (Illumina) and sequenced using an Illumina HiSeq 2000 platform. The resulting sequencing reads (100 bp) were assembled using Trinity (2) and annotated by BLAST (3) against the NCBI nonredundant protein database. We previously reported the genome sequence of *Diabrotica virgifera virgifera virus 1*, (DvvV1), an iflavirus (1), and *Diabrotica virgifera virgifera virus 2* (DvvV2), an unclassified novel ssRNA virus (4). Here, we report the sequence of the third small RNA virus discovered from WCR.

The genome of this virus, tentatively named *Diabrotica virgifera virgifera virus 3* (DvvV3), has 13,182 nucleotides (nt), a polyA tail, and is AT-rich (66.24% AT). The genome contains two open reading frames (ORFs), ORF1 from nt 676 to 9189 and ORF2 from nt 9235 to 12453, as well as a 674-nt 5′ untranslated region (UTR) and 726-nt 3′ UTR. The intergenic region (IGR) is only 45 nt in length. The 5′ end rapid amplification of cDNA ends (SMARTer RACE cDNA amplification kit, Clontech) did not result in identification of an additional sequence. The majority of the DvvV3 sequence was confirmed by reverse transcription-PCR using SuperScript Ш (Life Technologies, Inc.). Sequences of DvvV3 were detected in field-collected samples from the United States and Europe, and in the transcriptome of an inbred, nondiapausing laboratory culture of *Diabrotica virgifera virgifera* maintained at the USDA-ARS North Central Agricultural Research Laboratory, Brookings, South Dakota, USA.

ORF1 encodes a nonstructural polyprotein of limited sequence homology to the polyprotein of *Rosy apple aphid virus* (5) (ABB89048.1; 23% amino acid [aa] identity, 23% sequence coverage), and a nonstructural polyprotein of *Nilaparata lugens C virus* (6) (AIY53985.1; 24% aa identity, 18% sequence coverage). Three protein domains were identified: Macro_poa1p_like domain (cd02901, aa 74 to 175), RNA_helicase (pfam00910, aa 1008 to 1110), and RNA-dependent RNA polymerase (RdRP_1) domain (pfam00680, aa 2249 to 2797). An RNA protease motif (GxCG) is located at aa 2095 to 2098. ORF2 did not show significant similarity to any known proteins but did show a weak similarity to a partial capsid protein of *California sea lion norovirus 1* (*Norovirus*, *Caliciviridae*) (7) between aa 194 to 367, suggesting that ORF2 encodes a structural polyprotein. Although the genome of DvvV3 is dicistrovirus-like, the protein sequences encoded are divergent from these and other insect viruses, which is suggestive of a new type of positive-sense ssRNA virus.

### Accession number(s).

The DvvV3 genome sequence was deposited in GenBank under accession number KY200663.
